
JOURNAL INFORMATION
==============================
NLM Title Abbreviation: Pregnancy (Hoboken)
Journal ID: Pregnancy (Hoboken)
NLM Journal ID: 9918994890806676
Journal ID: PMF2

Pregnancy

EISSN: 2997-9684
Publisher: Wiley

ARTICLE INFORMATION
==============================
PMCID: PMC13344675
DOI: 10.1002/pmf2.70154
Article ID: PMF270154
Article version: 1
Subjects: Abstracts, Oral Concurrent Session 8

oral concurrent session 8 Hypertension

Abstracts 82 – 89 FRIDAY February 13, 2026 8 AM – 10 AM Summit 211 – 214 moderators Eugene Chang, MD Heather Lipkind, MD, MS

Electronic publication date: 2026 Feb 9
Publication date: 2026 Jan
Volume: 2
Issue: Suppl 1
Electronic Location ID: e70154
Accepted 2025 Oct 16
Copyright: © 2026 Society for Maternal‐Fetal Medicine. Pregnancy published by Wiley Periodicals LLC on behalf of the Society for Maternal‐Fetal Medicine.
License: This is an open access article under the terms of the http://creativecommons.org/licenses/by/4.0/ License, which permits use, distribution and reproduction in any medium, provided the original work is properly cited.
License URL: https://creativecommons.org/licenses/by/4.0/

Figure count: 0
Table count: 0
Page count: 1

source-schema-version-number: 2.0
cover-date: January 2026
details-of-publishers-convertor: Converter:WILEY_ML3GV2_TO_JATSPMC version:6.8.3 mode:remove_FC converted:07.07.2026

==============================

### Oral Concurrent Session 8

### Hypertension

Abstracts 82 – 89

FRIDAY
February 13, 2026
8 AM – 10 AM
Summit 211 – 214

MODERATORS
Eugene Chang, MD
Heather Lipkind, MD, MS
